# Comparative Study of Loading of Anodic Porous Alumina with Silver Nanoparticles Using Different Methods

**DOI:** 10.3390/ma6010206

**Published:** 2013-01-14

**Authors:** Sanjay Thorat, Alberto Diaspro, Alice Scarpellini, Mauro Povia, Marco Salerno

**Affiliations:** 1 Department of Nanophysics, Istituto Italiano di Tecnologia, via Morego 30, Genova I-16163, Italy; E-Mails: sanjay.thorat@iit.it (S.T.); alberto.diaspro@iit.it (A.D.); 2 University of Genova, viale Causa 13, Genova I-16145, Italy; 3 Department of Nanochemistry, Istituto Italiano di Tecnologia, via Morego 30, Genova I-16163, Italy; E-Mails: alice.scarpellini@iit.it (A.S.); mauro.povia@iit.it (M.P.)

**Keywords:** anodic porous alumina, nanoparticles, silver, filling factor, elution

## Abstract

Three different routes were used to infiltrate the pores of anodic porous alumina templates with silver nanoparticles, selected as an example of a bioactive agent. The three methods present a continuous grading from more physical to more chemical character, starting from *ex situ* filling of the pores with pre-existing particles, moving on to *in situ* formation of particles in the pores by bare calcination and ending with *in situ* calcination following specific chemical reactions. The resulting presence of silver inside the pores was assessed by means of energy dispersive X-ray spectroscopy and X-ray diffraction. The number and the size of nanoparticles were evaluated by scanning electron microscopy of functionalized alumina cross-sections, followed by image analysis. It appears that the best functionalization results are obtained with the *in situ* chemical procedure, based on the prior formation of silver ion complex by means of ammonia, followed by reduction with an excess amount of acetaldehyde. Elution of the silver content from the chemically functionalized alumina into phosphate buffer saline has also been examined, demonstrating a sustained release of silver over time, up to 15 h.

## 1. Introduction

Nowadays, nanostructures are commonly applied in materials and devices used in many diverse areas of science and technology, from electro-optical and mechanical engineering to the life sciences, from chemical and environmental sensors to medical diagnostics. The nanoscale in materials can be reached by either assembling nanoparticles (NPs) into ordered patterns or random ensembles, as, e.g., inside resin nanocomposites, or by using extended nanostructured matrices formed after self-assembly during the respective physical–chemical fabrication. Sometimes, both nanostructure types, *i.e.*, particles and matrix, can be combined and interact with each other, giving rise to possible operating functions. This is, for example, the case of anodic porous alumina (APA [1]) as the matrix phase. APA is often used as a template in which case its porous structure works as a kind of multiple high-density parallel reactor. In fact, each pore can act as a vessel, allowing peculiar conditions to be maintained inside, thus providing the environment for a controlled physical–chemical process [2]. Whereas advanced biochemistry could be carried out in APA at the molecular scale, for example, with biopolymers, to date, the most advanced applications of APA templates are still limited to masks for the bottom deposition of NPs [3] or molds for the fabrication along the pores of nanowires or nanotubes, made either of metals [4], polymers [5] or oxides [6]. The use of APA as a template is normally followed by its selective dissolution in acidic aqueous solutions to set the formed nanostructures free of their mold. 

In previous works, we used the surface features of an APA substrate to replicate them into a metal by simple coating [7]. In this work, we present the realization of an APA substrate with NPs of silver (Ag) taking place in the pores. Ag was selected as the NPs material since it has high physical–chemical stability, is inexpensive and is a well-known biocide [8]. The loaded Ag can be subsequently released over time, providing a potential use of the loaded APA as an antibacterial drug release carrier. This application of APA has already been demonstrated [9] and advocated to be potentially useful in many medical applications, from orthopedics to dentistry [10]. Here, we report the details about three different methods that can be used to load APA with Ag NPs and the respective characterization. Finally, for the best functionalized APA substrate, the potential action as a drug carrier is also evaluated, by assessing the Ag elution when submerged in phosphate buffer saline (PBS) solution at room temperature (RT) over time, up to 24 h. 

## 2. Results and Discussion

**Figure 1:**
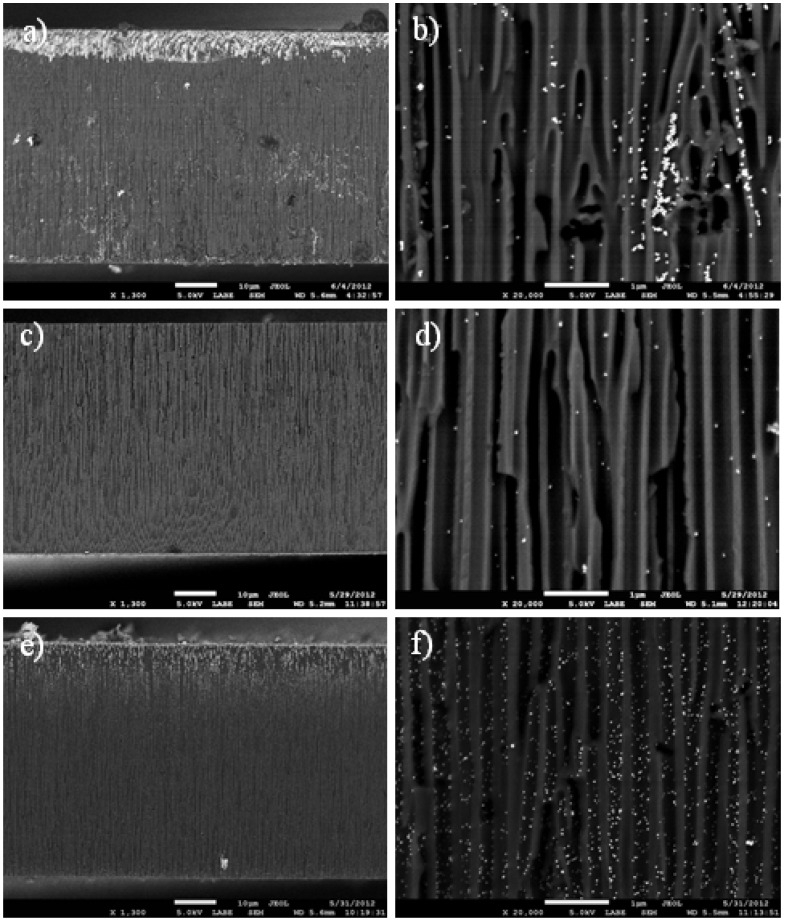
Scanning electron microscope (SEM) images of Ag-loaded anodic porous alumina (APA) substrates prepared from different methods; (**a**)–(**b**) direct filling with commercial nanoparticles (NPs); (**c**)–(**d**) physical method; (**e**)–(**f**) chemical method. Magnification is 1300× for the left column images (**a**), (**c**) and (**e**), and 20,000× for the right column images (**b**), (**d**) and (**f**). (**b**) is not representative of the typical surface, but is rather selected on purpose in a region of high NPs aggregation.

In Figure 1, typical scanning electron microscope (SEM) images of the samples prepared according to the three different loading methods are presented. On the left column, lower magnification images (1300×) show the whole cross-section for a best overview of the APA loading, whereas on the right column, higher magnification images (20,000×) allow evaluation of the number and size of individual NPs deposited inside the pores. It appears clearly that for the NPs, filling (Figure 1a) an inhomogeneous loading of Ag occurs, mainly concentrated at one side of the APA membrane. This is actually the top face of the APA sitting in the beaker containing the NPs solution. Obviously, the penetration of NPs into the APA occurs preferentially on this side, whereas the bottom side is hindered to access by the NPs in solution, as their diffusion into the pores is not favored there. At the same low magnification, for the physical method (Figure 1c), a much more uniform distribution of particles is obtained, though with lower concentration on average. Indeed, at a closer look (compare Figure 1b,d), only very few NPs appear in this case. Actually, the close-up in Figure 1b is not really representative of the typical NPs filling sample: in most images, almost no particles at all appeared, and only in two of them, like the one presented here, clustered particles appeared instead. For calculation of the actual NPs size, we used images similar to that of Figure 1b. However, for evaluation of the particle coverage across the section, we averaged all the images taken at different positions, according to what is described in Section 3.3. 

In the chemical method case (Figure 1e,f), again, some large-scale non-uniformity appears, with denser Ag presence in the 10–15 µm in-depth section closer to the top face, but with much better local uniformity in the middle-depth region of the APA, and at a comparatively higher level of NPs content (compare Figure 1d,f). The slight in-depth non-uniformity appearing here can also be justified by the chemical reactions involved in NPs formation being favored closer to the top face, similar to the NPs filling. However, in this case, much weaker restrictions due to diffusion appear, as the diffusing objects are here ionic or molecular species, rather than relatively large (≥10 nm size scale) NPs. 

**Figure 2:**
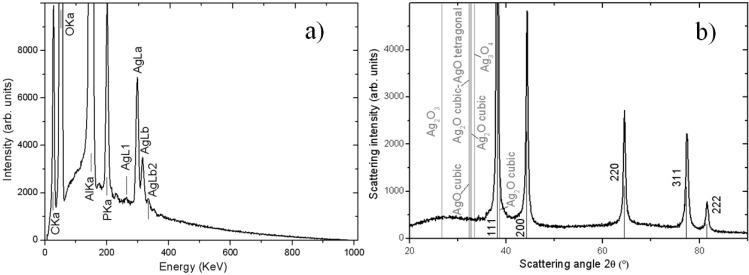
(**a**) Energy-dispersive X-ray microanalysis (EDS) spectrum of one APA substrate Ag-loaded with the chemical method; (**b**) X-ray diffraction (XRD) spectrum for the same sample.

Since the backscattered electrons contrast in SEM was not sufficient to assess the chemical nature of the NPs, on the high magnification images we also carried out energy-dispersive X-ray microanalysis (EDS). One typical such spectrum is reported in Figure 2a, for the case of the chemical loading method. As expected, among the different elemental species present, the contributions of aluminum (Al) and oxygen (O) from the APA frame are dominant. Additionally, we observed the presence of carbon (C), phosphorus (P) and Ag, as expected. C is there as the top conducting layer deposited on purpose on the samples (see Experimental section), in addition to being a common contaminant from organic compounds, resulting from solvents in the residual atmosphere of the SEM vacuum chamber. P is probably there as a contaminant from the fabrication itself of the APA templates, which at the industrial level are usually obtained by anodization of the Al in aqueous phosphoric acid electrolyte. This process is known to result in the trapping of some phosphate ions (3% atoms here, with respect to 6% reported in [11], probably for so-called hard anodization). However, what is most important for us is the presence of Ag coming from our loading process. 

In addition to EDS, for one Ag-loaded APA sample of each type, also powder X-ray diffraction (XRD) measurements were carried out, and the results for the same sample (chemical loading method) as for the EDS are shown in Figure 2b. This type of analysis further confirmed the composition of the NPs being Ag. In particular, for all the loading methods, the XRD spectral features matched closely to the fingerprint of metallic Ag, with the peaks of the different orientations of cubic Ag crystals appearing, as contained in the Card No. 03-065-2871 of the JCPDS database (see labels in Figure 2b). Note, that there is no presence of peaks from APA, which is present in amorphous form, giving rise to the shallow broad band in the 20°–35° range. Only upon heating up to ≥700 °C, APA is known to partly crystallize to the γ phase [12].

In addition to confirming the results of EDS in terms of the presence of Ag, the XRD data provide extra information about the structure of the Ag. In Figure 2b, along with the spectral positions (vertical black lines) corresponding to the cubic phase of metallic Ag, the positions (gray lines) corresponding to the dominant peaks of the different Ag oxides are also reported (see the labels in Figure 2b). Clearly, no match of any oxide-specific line is there with the experimental spectrum, showing that all the loaded NPs were completely metallic soon after deposition. We can conclude that the Ag in the NPs is not passivated by oxidation in the short time (scale of h), and their bioactive potential is therefore preserved.

From the SEM images, a statistic of the NPs size and total relative area has been extracted, and the results are reported in Table 1. For the NP diameter, the mean and standard deviation are used as the representative value and uncertainty, respectively. The surface coverage along the cross-sections of both the NPs and the APA pore voids has also been evaluated, and thus, it was possible to calculate the ratio of the two, which represents the coverage of the pore cross-sectional area by the NPs. This quantity is used here as a rough estimate of the volume filling (%) of the NPs inside the pores. The uncertainty assigned to the respective values (column ‘Pore coverage by NPs’ in Table 1) is calculated after the propagation of errors, by considering as the best estimate the most probable error of the value resulting from summing the relative errors in quadrature, Δ(a/b) = <a/b>·√[(Δa/<a>)^2^+(Δb/<b>)^2^], where the <> signs represent the mean values.

From the numbers in Table 1, it is clear that the Ag NPs fabricated *in situ* by both the physical and the chemical methods are smaller than the commercial NPs, (approximately 35 and 25 nm diameter, respectively). The method that provided the highest filling was the chemical one (NPs pore coverage ~6.1%). In this respect, the NPs filling scores second, with ~2.6% coverage *versus* the ~1.6% of the physical method. Additionally, the high spread of the NPs filling method, with a coefficient of variation (ratio of mean to standard deviation) of ±71%, confirms that in these samples, the distribution of NPs throughout the APA thickness is less uniform than for the physical and, especially, the chemical method, with a coefficient of variation of ±62 and ±39%, respectively. Thus, in both typical level (the highest mean) and uniformity (the lowest spread), the method that provides the best loading appears to be the chemical one.

**Table 1:** Summary of morphological parameters extracted from the SEM images similar to those in Figure 1b,d,f.

Loading method	NPs diameter (nm)	Image coverage (%)		Pore coverage by NPs (%)
		NPs	Pores	
NPs filling	50 ± 11	1.4 ± 1.0	54 ± 12	2.6 ± 1.9
Physical	36 ± 25	0.8 ± 0.5	49 ± 14	1.6 ± 1.1
Chemical	24 ± 10	2.8 ± 1.1 ^#^	46 ± 10	6.1 ± 2.7

^#^ after ANOVA analysis, the NPs image coverage by chemical loading appears significantly much different (**) from the physical loading value and also different (*) from the NPs loading value.

In order to understand if the above mentioned differences are statistically significant, on the raw NPs coverage data (*N* = 6), we performed a one-way analysis of variance (ANOVA) with Tukey pairs test for comparison between the means. The significance levels selected were α = 0.01 (“much different”, **) and α = 0.06 (“different”, *). According to these levels, the chemical loading value appeared much different from (higher than) the physical loading value and different from (higher than) the NPs filling value, whereas there was no statistically significant difference between NPs filling and physical loading. Actually, the weaker “different” threshold has been set to 0.06, instead of the more common 0.05, for better discrimination of the above groups of experimental datapoints. In fact, the Tukey tests showed a probability that the means of the chemical loading and NPs filling data are different by chance—yet belonging to the same parent distribution—of *p* = 0.055, which would result in a “no difference” response with an α = 0.05 threshold. However, using this threshold, one would miss the point that the data from the remaining pair of loading methods (NPs filling–physical loading) is *much* less different (*p* = 0.423).

**Figure 3:**
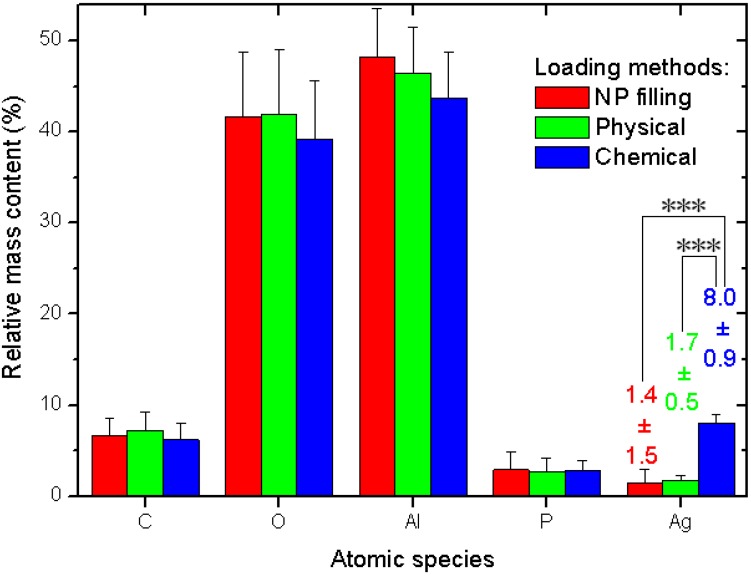
Results of EDS analysis of Ag-loaded APA from different methods.

The quantitative data and related statistics obtained from all the EDS measurements are reported in Figure 3. The plot represents the percent mass composition of the Ag-loaded APA substrates for different elemental species, with the respective mean (bar height) and standard deviation (error bar, *N* = 6). As mentioned in the description of Figure 2a, obviously Al and O are the dominating species. When converted into the respective atomic percent compositions, the ratio of the two (No. (atoms O)/No. (atoms Al)) gives the stoichiometric value of 1.50 ± 0.03, as expected for the alumina material (Al_2_O_3_) of which APA is made. 

Our interest is rather focused on Ag. Whereas the other species show roughly constant contents—within the errors—for all the three different loading methods, the Ag loading shows significant difference between the methods. The NPs filling and physical method exhibit similar values, again, with a much higher spread for the former (see the numerical values label in Figure 3). However, the chemical method shows a much higher value than both the other methods, with a difference that, after the ANOVA pairs comparison with Tukey test, appears in both cases statistically significant at the highest threshold level selected of α = 0.001, (very much different, ***), whereas the pair NPs filling–physical loading does not appear different at even the weakest level of α = 0.1. 

We now compare the apparent efficacy of Ag filling of the different methods resulting from the SEM images and the EDS data. From the SEM analysis, it appears that the NPs filling method scores higher than the physical method, whereas the contrary appears for the EDS analysis. In fact, the relatively high uncertainty in the resulting numbers makes the NPs filling and the physical method results not significantly different from the statistical viewpoint for both types of analysis. However, in both cases, the chemical method shows the highest filling. When this is normalized to the value of the respective lowest filling method, it appears to be higher in a ratio of 3.8× from SEM data and 5.7× from EDS data. Actually, since the SEM values are obtained from a surface coverage rather than a volume filling, a first rough correction of these data would require them to be raised to the power 3/2, which would give 7.4×. This value is shifted to higher numbers in the direction of the EDS value and beyond that. Obviously, given the complex structure of the hexagonal pattern of APA pores, such a simple scaling with the spatial dimensions is probably only a crude approximation of the real behavior, also due to the finite depth of focus of the SEM images. Therefore, some intermediate value would be more correct for the SEM data and closer to the EDS ones.

**Figure 4:**
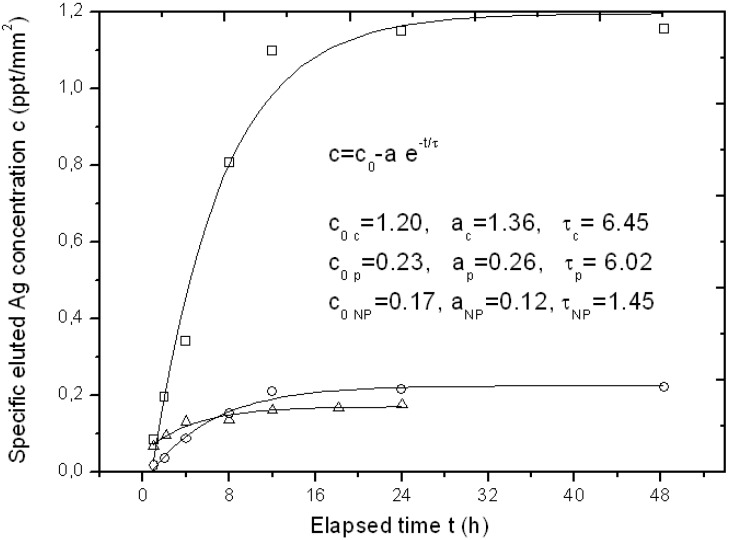
Typical curves of eluted Ag concentration per unit surface area from APA loaded with the three different loading methods up to the first two days, with respective fitting parameters to an exponential growth model. The symbols legend for experimental datapoints is as follows: Squares (□) are used for chemical loading, circles (○) for physical loading and triangles (Δ) for NPs filling.

For some specimens of the APA template Ag-loaded with the three different methods, we also tested its function as a possible carrier for drug delivery of the bioactive Ag. Typical representative results of Ag elution as measured by inductive coupled plasma optical emission spectrometry (ICP-OES) over a time of up to two days have been reported in Figure 4. 

Since the APA specimens were of a different surface area, the amount of eluted Ag concentration was normalized to the approximate surface area of the APA piece, obtaining a specific concentration c (in ppt/mm^2^). In Figure 4, three representative profiles resulting from the APA samples loaded with the three different methods are shown. The typical sigmoidal profile of elution from a drug-delivery carrier appears, with an initially linear increase of released Ag over time, and a final saturation level c_0_. The experimental datapoints have been fitted to a simple model of exponential growth with time, c = c_0_−a·e^−t/τ^, where c_0_ represents the final specific concentration reached after elution is completed, a is a constant factor and τ is a characteristic rising time. All fitting results are reported in the inset of Figure 4. Indices c, p and NP stand for chemical loading, phisical loading and NPs filling methods, respectively. It clearly appears that the type of sample with the highest release is the chemical loaded APA, as expected (c_0 c_ ~ 1.2 ppt/mm^2^), whereas both the NPs filled and the physical loaded APA give roughly six-fold lower final elution levels. The c_0_ levels for the two latter methods (c_0 NP_ and c_0 p_) appear similar even if the loading level was higher for the NPs filling: obviously the Ag loaded into the APA with the latter method is not easily released upon immersion of the sample in a liquid, as it formed a compact layer on top of the APA rather than dispersed and loosely bound Ag NPs. 

From Figure 4, the characteristic time constant of the elution can also be derived. It appears that both the *in situ* methods that provide dispersed Ag NPs, *i.e.*, the chemical and the physical loading, have similar time scales (τ_c_ ~ τ_p_ = 6–6.5 h), whereas the NPs filled APA reaches the final state c_0_ in a shorter time (τ_NP_ ~ 1.5 h), since all the little Ag available for release is already facing the top surface in direct contact with the liquid. 

## 3. Experimental Section 

### 3.1. Materials

The APA substrates used were commercially available Anodisc membranes, with given specifications of 200 nm pore diameter and 60 μm thickness (Whatman Ltd., UK). For the direct filling method, colloidal silver nanoparticles were purchased, suspended in triethylene glycol monomethyl ether, with a nominal 30–35 wt % concentration and <50 nm diameter (product No. 736473, Sigma Aldrich, Italy). For the other two loading methods, Ag nitrate (AgNO_3_) was used as the Ag supplier (product No. 11414, Alfa Aesar, Germany). A 25% solution of NH_3_ (ammonia, product No. 1054321000) and CH_3_CHO (acetaldehyde, product No. 8000040500) were also used (both from Merck Chemicals, Germany) as a complexing and reducing agent, respectively. All chemicals were used as received, without any further purification. 

### 3.2. Loading Methods

The three different methods used have been called, briefly, “NPs filling” and “physical” and “chemical” loading. All the experiments were carried out in air at RT, except the NPs filling experiment, as the commercial NPs had to be handled in a glove-box. To compare the results of each method, we have fixed the Ag content of the respective solutions to 5 wt % and the APA membrane impregnation time to 30 min for all methods. The detailed procedures used for each method are described in the next paragraphs. 

#### 3.2.1. NPs Filling 

This method is *ex situ* in character, since we used commercial Ag colloidal NPs that were available before being inserted into the APA pores. The NPs solution was first diluted using the same solvent as the mother solution (triethylene glycol monomethyl ether) to reach the target reference value with a Ag concentration of 5 wt %. The APA membranes were immersed into the solution for the impregnation step. After that, they were removed from the bath, and the excess surface solution was washed away with deionized (DI) water. Then, both opposite membrane surfaces were gently touched with debris-free tissue lens paper, and finally, the substrate was dried in air at 60 °C for 2 h. 

#### 3.2.2. Physical Loading

AgNO_3_ solution (also 5 wt % in Ag) was prepared, and the APA membrane was dipped into it for 30 min at RT. Due to capillary interaction, the pores were filled with the AgNO_3_ solution. The excess solution was then removed by nitrogen blowing and by touching with lens paper. Then, the AgNO_3_ filled APA template was dried at 110 °C for 1 h to evaporate the solvent, and it was finally calcined in a furnace at 500 °C for 3 h, with a ramping rate of 3 °C/min. 

#### 3.2.3. Chemical Loading

Same as for the physical loading method, an AgNO_3_ solution (5 wt % of Ag) was first prepared. Second, 3 wt % ammonia was added dropwise into 20 mL AgNO_3_ solution, for the generation of a precursor solution of Ag(NH_3_)_2_^+^ complex ions, until the newly generated precipitate dissolved. Third, 3 mL precursor solution was taken into a glass vial of 30 mL, and a piece of APA was dipped into it and allowed to impregnate. After that, under the vibration of the vial, 40 wt % acetaldehyde solution was added dropwise for *in situ* reduction. An excess amount of acetaldehyde was finally added to ensure complete reduction. After adequate mixing, the vial was placed in a water-bath at 50 °C for 30 min. The templates were carefully washed 3 times with DI water, touched with lens paper and finally dried in air at 60 °C for 2 h.

### 3.3. Characterization Techniques

The Ag-loaded APA membranes were cut with a scalpel, and the cross-sections were coated with a ~5 nm layer of C in order to make SEM possible without artifacts due to electrostatic charging after electron beam impingement. Field-emission SEM was carried out on a JSM-7500F (Jeol, Japan) at an accelerating voltage of 5 kV and typical magnifications of 1300× and 20,000×. We preferentially used a backscattered electron detector, which provides the best direct contrast between the APA sidewalls and the high atomic weight of the metal NPs. For each sample, six images at the highest magnification were taken at different depths across the section (two at ~1/4 depth, two at ~1/2 depth and two at ~3/4 depth). Those images have been treated by segmentation according to the standard method of brightness thresholding by means of the freeware program Gwyddion 2.25 (Cszech Metrology Institute, Cszech Republic), and the respective quantities extracted have been averaged. For assessment of the statistical significance of the apparent differences, the raw data (*N* = 6) of NPs image coverage have been analyzed with one-way ANOVA and the means compared with the Tukey test. The two significance levels of α = 0.06 (statistically “different”) and α = 0.01 (statistically “much different”) have been selected as the thresholds for the best segmentation of the three groups of data (see Section 2. Results and Discussion). 

The SEM imaging was integrated with EDS. The EDS raw data were also subjected to ANOVA (*N* = 6) with the Tukey pairs test at the significance levels of α = 0.1, 0.01 and 0.001. This chemical analysis was further supplemented by X-ray diffraction (XRD) carried out with a Smartlab 9 kW diffractometer (Rigaku, Japan) equipped with a copper rotating anode. The X-ray source was operated at 40 kV and 150 mA with a Gobel mirror to make the beam parallel and to remove CuKβ (1.392 Å). We did not use a monochromator, so the data was obtained using CuKα1(1.544 Å) and CuKα2 (1.541 Å). Beam size was 0.8 × 5 mm^2^, and the measure was performed over the 2θ range from 5° to 90°, with a step of 0.05° and a scan speed of 3°/min. The diffraction pattern was analyzed by the PDXL software (Rigaku), and pure metallic Ag was identified by the JCPDS 03-065-2871 card.

Finally, for some specimens of APA loaded with the different methods (*N* = 2 for the NPs filling, *N* = 2 for the physical loading and *N* = 3 for the chemical loading), the respective elution of Ag NPs into PBS aqueous solution (1×) at RT has been evaluated. The differently sized pieces of Ag-loaded APA discs were dipped each in a beaker with 1.5 mL PBS. At pre-determined time instants of 1, 2, 4, 8, 12, 24 and 48 h, 250 µL of solution was removed by micropipette after appropriate remixing (flushing in and out the tip 5 times). The taken solution was set to Ag digestion by means of aqua regia (1:3 HNO_3_:HCL) overnight and diluted to 10 vol % with DI water. The subsequent morning, the solution was analyzed with ICP-OES carried out with an iCAP 6000 Series spectrometer (Thermo Scientific, Germany) after proper instrument calibration. As a result, the relative amount of selected elemental chemical species was investigated and the content of Ag was found, expressed in parts per trillion (ppt).

## 4. Conclusions 

We have presented three different methods for loading APA with Ag NPs. The system has been characterized with different techniques to make sure that the NPs grown inside the template are indeed metallic Ag. Our experimental results show that the different methods provide a different filling factor in the range of 1% to 8%, approximately. The method allowing for the highest filling, called the chemical method, is based on the use of ammonia and acetaldehyde as a complexing and reducing agent, respectively, for which a practical recipe is provided. In particular, the highest filling value obtained with this method is also accompanied with good NPs distribution uniformity and the smallest NPs size (mean diameter ~25 nm). Both the significant mass loading and the small NPs size should make this type of sample relevant from the pharmaceutical point of view for the potential effect of the Ag content. To demonstrate the potential application of our samples in this field, we have finally investigated the elution of Ag from all the three types of Ag-loaded APA in a typical solution used in the biophysical context, which is PBS. The obtained results confirm that the best method of APA loading with Ag NPs also allows for the best performing subsequent elution after NPs diffusion in PBS, with sustained NPs release over a time of up to ~15 h, which is significant for the possible use of the loaded NPs for pharmaceutical applications.
